# Childhood Blood Lead Levels and Symptoms of Attention Deficit Hyperactivity Disorder (ADHD): A Cross-Sectional Study of Mexican Children

**DOI:** 10.1289/ehp.1510067

**Published:** 2015-12-08

**Authors:** Siying Huang, Howard Hu, Brisa N Sánchez, Karen E. Peterson, Adrienne S. Ettinger, Héctor Lamadrid-Figueroa, Lourdes Schnaas, Adriana Mercado-García, Robert O. Wright, Niladri Basu, David E. Cantonwine, Mauricio Hernández-Avila, Martha María Téllez-Rojo

**Affiliations:** 1 Dalla Lana School of Public Health, University of Toronto, Toronto, Ontario, Canada; 2 University of Michigan School of Public Health, Ann Arbor, Michigan, USA; 3 Yale School of Public Health and Medicine, New Haven, Connecticut, USA; 4 Instituto Nacional de Salud Pública, Cuernavaca, Morelos, México; 5 Instituto Nacional de Perinatologia, Mexico City, Mexico; 6 Icahn Mount Sinai School of Medicine, New York City, New York, USA; 7 Faculty of Agricultural and Environmental Sciences, McGill University, Montreal, Québec, Canada; 8 Brigham & Women’s Hospital, Harvard Medical School, Boston, Massachusetts, USA

## Abstract

**Background:**

Previous studies suggest that blood lead levels are positively associated with attention deficit/hyperactivity disorder (ADHD) and ADHD-symptoms in children. However, the associations between lead exposure and ADHD subtypes are inconsistent and understudied.

**Objective:**

The objective of this study was to explore the association of low-level concurrent lead exposure with subtypes of ADHD symptoms in 578 Mexican children 6–13 years of age.

**Methods:**

We measured concurrent blood lead levels using inductively coupled plasma mass spectrometry (ICPMS). We administered the Conners’ Rating Scales-Revised (CRS-R) to mothers to evaluate their children’s ADHD symptoms. We used imputation to fill missing values in blood lead levels and used segmented regression models adjusted for relevant covariates to model the nonlinear relationship between blood lead and ADHD symptoms.

**Results:**

Mean ± SD blood lead levels were 3.4 ± 2.9 μg/dL. In adjusted models, a 1-μg/dL increase in blood lead was positively associated with Hyperactivity and Restless-Impulsivity scores on the CRS-R scale and Hyperactivity-Impulsivity scores on the CRS-R scale of the Diagnostic and Statistical Manual of Mental Disorders, 4th Edition, but only in children with blood lead level ≤ 5 μg/dL. Blood lead was not associated with Inattentive symptoms or overall ADHD behavior.

**Conclusions:**

In this population of Mexican children, current blood lead level among children with low exposure (≤ 5 μg/dL) was positively associated with hyperactive/impulsive behaviors, but not with inattentiveness. These results add to the existing evidence of lead-associated neurodevelopmental deficits at low levels of exposure.

**Citation:**

Huang S, Hu H, Sánchez BN, Peterson KE, Ettinger AS, Lamadrid-Figueroa H, Schnaas L, Mercado-García A, Wright RO, Basu N, Cantonwine DE, Hernández-Avila M, Téllez-Rojo MM. 2016. Childhood blood lead levels and symptoms of attention deficit hyperactivity disorder (ADHD): a cross-sectional study of Mexican children. Environ Health Perspect 124:868–874; http://dx.doi.org/10.1289/ehp.1510067

## Introduction

An increasing trend in emotional and behavioral disorders in early childhood has been observed in the last 10 years (Boyle et al. 2011). Attention deficit/hyperactivity disorder (ADHD) is a psychiatric disorder that is characterized by attention deficits, hyperactivity, and impulsivity and is one of the most common emotional and behavioral disorders among children, with a worldwide prevalence estimated at about 5% (Polanczyk et al. 2007). Genetic inheritance is believed to play a major role in the etiology of ADHD. Twin studies have shown a high concordance of inattentiveness, with heritability estimated to range from 70% to 95% (Greven et al. 2011; Willcutt et al. 2000b); however, twin studies have not shown a high concordance for hyperactive or impulsive behaviors (Greven et al. 2011; Paloyelis et al. 2010; Willcutt et al. 2000a).

Recent studies suggest that environmental toxicants contribute to the risk of ADHD (Polanska et al. 2012; Tarver et al. 2014). Several case–control studies (Braun et al. 2006; Kim et al. 2013; Sánchez-Villegas Mdel et al. 2014; Wang et al. 2008) and cross-sectional studies (Froehlich et al. 2009; Ha et al. 2009; Nicolescu et al. 2010; Roy et al. 2009) have demonstrated associations between elevated blood lead levels and general ADHD behavior. However, only a few studies had examined lead exposure with regards to subtypes of ADHD. Some have shown that lead exposure is associated with hyperactive-impulsive behaviors, but not with attention problems (Boucher et al. 2012a; Nigg et al. 2008, 2010b). The mechanism of lead contributing to the risk of ADHD, especially with regard to the different behavior subtypes, remains unclear.

The objective of this study was to explore the association between concurrent lead exposure and the prevalence of ADHD behaviors in environmental lead–exposed Mexican children 6–13 years of age, with regard to three subtypes: hyperactivity, inattention, and the combination of hyperactivity and inattention.

## Materials and Methods

### Study Population

The study population was part of two birth cohorts of mother–infant pairs enrolled in Mexico City, Mexico, starting in 1997 and 2001, as part of the parent project known as the Early Life Exposure in Mexico to ENvironmental Toxicants (ELEMENT) study (Téllez-Rojo et al. 2006). Pregnant women were initially recruited from prenatal clinics of the Mexican Social Security Institute in Mexico City, which serves a low- to middle-income population formally employed in the private sector. Potential participants were excluded if they exhibited any factor that could interfere with maternal calcium metabolism or a family or personal history of a calcium-related disorder, had intention not to breastfeed, serious pregnancy-related conditions, taking corticosteroid medications, or were a single parent. They were later excluded if their child had any of the following at birth: gestational age < 37 weeks, birth weight < 2,000 g, Apgar score at 5 min of ≤ 6, admittance to the NICU (neonatal intensive-care unit), or a serious birth defect. Initial follow-up of the 1,079 children born to these two cohorts assessed growth and neurodevelopment at 6-month intervals until 5 years of age. Children were invited to the Department of Developmental Neurobiology at the Instituto Nacional de Perinatología for an additional follow-up visit between 2008 and 2011; 622 (58%) participated in the follow-up study when they were 6–13 years of age. Of those, 39 subjects were newly enrolled younger siblings of the study subjects from the original cohorts and were therefore excluded because they were missing baseline information. The remaining 583 (93.7%) mother–child pairs participated in the behavioral assessment visit as part of the follow-up study. An additional 5 subjects with birth weight < 2,000 g were excluded, leaving a total of 578 subjects in this analysis. The research was approved by the institutional review boards of the National Institute of Public Health of Mexico and all of the participating hospitals and institutions. Written informed consent and/or assent were obtained from all participants.

### Lead Exposure Measurements

A total of 166 of 578 children who participated in the study declined phlebotomy, leaving 412 children in the study with blood samples. Whole blood was collected from peripheral veins into trace metal–free tubes after sanitation at the lancet site. Blood samples were shipped to the University of Michigan and maintained at –80°C until analysis. A total of 342 samples were analyzed in the ISO (International Organization for Standardization)–designated clean room to minimize the contamination possibilities in University of Michigan Department of Environmental Health Sciences Metals Laboratory using inductively coupled plasma mass spectrometry (ICPMS) (Agilent 7500c; Agilent Technologies, Palo Alto, CA). Accuracy and precision were estimated by use of certified reference materials (Institut National de Santé du Québec; INSPQ, QMEQAS09) as well as replicated samples. The average detection limit was 0.03 μg/dL. The overall accuracy was 88.2% ± 9.5%. As part of the quality control step, 70 blood samples including 64 duplicates were analyzed at the Michigan Department of Community Health using a similar ICPMS approach with a detection limit of 1.3 μg/dL and all values above the limit of detection (LOD). The overall accuracy was 102.4% ± 2%. We performed cross-validation between the two laboratories in 64 samples and reached a consistency of 96.2% after removing two outliers.

### Behavioral Assessment

At the clinic visit, Conners’ Rating Scale–Revised (CRS-R) (Conners 2000) was administered to mothers to assess their children’s behavior. CRS-R examines seven types of behavior problems in children including: Oppositional, Anxious-Shy, Cognitive Problem/Inattention, Hyperactivity, Perfectionism, Psychosomatic, and Social Problems. It also contains four index scores: Conners’ ADHD Index; Conners’ Global Index (CGI): Restless-Impulsive; CGI: Emotional Lability; and CGI: Total. CRS-R also includes scales for the *Diagnostic and Statistical Manual of Mental Disorders, 4th Edition* (DSM-IV) diagnosis of ADHD: *a*) DSM-IV: Inattentive, *b*) DSM-IV: Hyperactive-Impulsive, and *c*) DSM IV: Total. The Spanish version of this scale has been validated in 139 Mexican first-grade children (internal reliability = 0.894) for the evaluation of ADHD (Ortiz-Luna and Acle-Tomasini 2006). We selected scales and index scores from CRS-R related to ADHD symptoms assessment: Cognitive Problem/Inattention, Hyperactivity, ADHD Index, Conners’ Global Index: Restless-Impulsive, and three subscales from DSM-IV symptoms subscales (Inattentive, Hyperactive-Impulsive, and Total). These scores reflect mainly hyperactivity, inattentiveness, or overall ADHD symptoms. All scales were standardized into T-scores. Higher scores indicate an increased likelihood that a child would meet diagnostic criteria for ADHD. A T-score of 65 indicates a “clinically significant problem” (Conners 2000).

### Other Information

ELEMENT has detailed information on maternal, child, and familial characteristics dating back to pregnancy. In addition, we collected updated questionnaire-based information on maternal marital status, parental education levels, and family socioeconomic status (SES) at recruitment for this child follow-up study. The socioeconomic questionnaire asked about the availability of certain items and assets in the home [number of light bulbs in the home, rooms in the house, bathrooms, cars, personal computer, water heater, electrical appliances (video/DVD player, washing machine, vacuum cleaner, toaster, microwave), and the type of house floor]. Point values were assigned to each item, and the SES level was calculated based on the sum of the points across all items.

### Statistical Analyses

Children’s blood lead levels were missing in 29% of study subjects because the test subjects or their parents declined phlebotomy. In addition, a few subjects (< 8%) had missing information on behavior tests and covariates of interests. We performed single imputation to replace all missing values. The imputation model assumed that the missing mechanism is missing at random (MAR) because declining phlebotomy, for example, is unlikely to depend on the children’s blood lead levels. The MAR assumption is more robust than the missing completely at random (MCAR) assumption because MCAR assumes that the probability of missing does not depend on any observed values (Little and Rubin 2002); however, in our data, declining phlebotomy occurred slightly more often among younger children (mean ± SD age for children who declined and consented: 8.7 ± 1.3 years vs. 9.2 ± 1.3 years). We used chained equations methods in R *mice* package (van Buuren and Groothuis-Oudshoorn 2011) to impute the missing values and used covariates and outcomes in the imputation models as recommended by Moons et al. (2006). Specifically, we used historical children’s blood lead levels at 12, 18, 24, 36, and 48 months and child’s sex, age, birth weight, and neurobehavior outcomes from the CRS-R scales, as well as parental information such as maternal age, educational level, marital status, SES level, smoking status during pregnancy, and paternal educational level to impute current children’s blood lead levels and other missing values. Children’s sex, maternal marital status, and smoking status during pregnancy were treated as binary variables, and the rest were treated as continuous variables. Briefly, the chained equations procedure first fills out the missing values with the variable mean and uses multivariate regression predictions to update imputed values until convergence. We used a predicted mean matching approach to select imputed values. That is, instead of using the predicted values from the regression, this method finds a value from the observed data that is close to the predicted value for replacement. This method has advantages of fast convergence, avoiding impossible values, and preserves the nonlinear relationship when model is mis-specified. We allowed 20 iterations in one imputation because the authors (Azur et al. 2011; van Buuren and Groothuis-Oudshoorn 2011) suggested that the convergence can be reached between 5 to 10 iterations. We examined the imputation convergence by plotting the means and standard deviations at each iteration over the number of iterations, as recommended (van Buuren and Groothuis-Oudshoorn 2011). We observed that the means and standard deviations were randomly distributed over iterations without any apparent increasing or decreasing trend. This demonstrates that the imputations took on plausible values.

We treated blood lead and behavioral assessment scores as continuous variables. We performed crude bivariate analyses between blood lead and CRS-R scales with locally weighted scatter plot smoothing (LOWESS) to examine potential nonlinear dose–response relationships. Blood lead did not show a linear relationship with behavior tests either before or after natural log–transformation. For a subset of the outcomes, LOWESS suggested that the relationship between blood lead and behavioral outcomes was increasing at first, followed by a plateau. Therefore, we used a segmented regression approach (Muggeo 2003) to *a*) identify breakpoints in the association (i.e., blood lead level at which tapering off of the association occurred), and *b*) assess the linear association between lead and behavioral outcomes between breakpoints.

Briefly, the segmented regression method assumes linear relationships between predictors and outcome within intervals, or segments, of the exposure distribution. The method estimates end points for said intervals, or “breakpoints,” from the data by iteratively updating the breakpoints and the exposure–outcome associations (i.e., slopes within the intervals) until the difference between the updated values and the last estimated values become close to 0 (convergence). The model itself cannot define the number of the breakpoints or initial values to begin the estimation procedure, and relies on the user for these specifications. We followed recommendations to use several initial values to assess sensitivity of the results to user input. We first used LOWESS methods to visualize nonlinear associations between children’s blood lead levels and CRS-R scales (see Figure S1). We observed apparent increasing trends from 0 to around 5–10 μg/dL on CRS-R scales of Hyperactivity, Restless-Impulsive, and CRS-R and DSM-IV scales of Hyperactive-Impulsive and Total and chose initial breakpoint values at 1, 8, and 15 μg/dL because these values were near the exposure levels where the relationships changed as observed in the LOWESS plots. For each of these initial values, we repeated the models at least 10 times and derived breakpoint and slope estimates from repeated models. We compared the breakpoint and slope estimates from repeated models within and across all the proposed initial values. For illustration, we present the results derived from these initial values, with each initial value run twice, in Table S1. When a true breakpoint exists in the data, the estimation of the breakpoint would be stable regardless of the initial values, and the confidence intervals (CIs) for the breakpoint would be relatively narrow. Otherwise, if the breakpoint does not exist, the model will fail to converge; yield different estimated breakpoints when different initial values are used; or give wide confidence intervals for the breakpoint. In these cases, the results rendered from segmented regressions do not have meaningful biological interpretations. As shown in Table S1, the breakpoint estimates stabilized between 5.0 and 5.4 on the CRS-R scales of Hyperactivity, CGI Restless-Impulsive, DSM-IV Hyperactive-Impulsive, and DSM-IV Total. The breakpoint estimates on the rest of scales either fell into extreme values (< 1 or > 20 μg/dL); the values of breakpoints were not consistent when using different initial values for iterations (i.e., on ADHD Index, the breakpoint switches between around 5 and 20 μg/dL); or the confidence intervals of breakpoints were relatively large (i.e., on DSM IV Total, the confidence interval ranges from 0.6 to 10.6 μg/dL, covering 29.1% of the lead exposure range), indicating the models could not find the apparent transitioning point for the relationship. Additionally, in the scales with low breakpoint values, the slope estimates for lower levels of blood lead tended to have large negative values, indicating steep decreasing slopes in the range of exposure where only a few observations were made. Given these criteria, we did not consider estimates from the segmented regression models to be biologically meaningful for the CRS-R Cognitive Problem/Inattention scale or ADHD Index, or the CRS-R DSM-IV Inattentive scale or Total score, and therefore used ordinary least square regression instead of segmented regression to estimate associations for these outcomes. Additionally, from residual diagnostic plots, we observed that outcomes had larger variance at the higher end of blood lead levels (data not shown); to correct this violation of the constant variance assumption in the model, we used robust standard errors derived using the “sandwich” formula, which are also implemented in the R *segmented* package (Muggeo 2008) by using the robust standard error option.

Based on previous work (Afeiche et al. 2011; Zhang et al. 2012), we selected maternal marital status, age, educational years, and SES, ever smoked during pregnancy, and the child’s age at behavioral testing, sex, and birth weight as *a priori* covariates. We also included paternal educational years because it was significantly correlated with child behavioral outcomes and blood lead.

As applicable (i.e., on scales CRS-R Hyperactivity, CGI Restless-Impulsive, and DSM-IV Hyperactivity), we conducted sensitivity analyses for segmented regressions using: *a*) fixed breakpoints of 5 μg/dL since this is the CDC level of concern for lead exposure; *b*) the segmented regression models with complete cases only (i.e., excluding observations with missing values), with breakpoints estimated, or fixed at 5 μg/dL or at the estimates obtained with the imputed data; *c*) a subset of data where the blood lead levels were measured in the department of Environmental Health Sciences laboratory at University of Michigan (EHS lab); and *d*) the imputed data set after including five subjects with birth weight < 2,000 g. The segmented models with fixed breakpoints were estimated using a piece-wise linear regression with a single knot at the fixed breakpoint.

All statistical analyses were performed in R software (R Core Team 2012). All significance testing was two-sided and statistical significance was determined at alpha level of 0.05.

## Results

A total of 578 subjects with various proportions of missing information (29% for blood lead levels) on major variables of interest were included in this analysis. Table 1 shows the characteristics of the study population in both incomplete and imputed data. We did not observe meaningful differences in the distributions of characteristics between the imputed and incomplete data. We report the statistics from imputed data here. Mean ± SD maternal age was 26 ± 5.4 years. Mean maternal education was 10.9 ± 2.8 years, which was similar to the mean paternal education of 10.6 ± 3.5 years. Most (73%) mothers were married, and only 18 (3%) reported having smoked during pregnancy. Average infant birth weight was 3,100 g (range, 2,000–4,400 g). Females accounted for 50% of children who ranged in age from 6.2 to 12.5 years at the follow-up visit for this study. The means of T-scores on the CRS-R subscales were slightly higher than the expected T-score means [50 (Conners 2000)]. About 12% to 20% of subjects on all selected scales exceeded the scores of 65, indicating possible clinically significant behavior problems (Conners 2000). Consistent with expectations, CRS-R scores were lower (indicating fewer symptoms) in children with higher maternal and paternal education and in children whose mothers were married, whereas average scores were higher (indicating more symptoms) among children whose mothers smoked during pregnancy and in five children with low birth weight (data not shown).

**Table 1:** Characteristics of parents and children participating in Mexican birth cohort study of lead exposure and behavior.

Characteristics^*a*^	Original data			Imputed data *n* = 578
	*n*	Percent missing	Mean ± SD or *n* (%)	Mean ± SD or *n* (%)
Behavior assessment
CRS-R
Cognitive Problem/Inattention	561	2.9	54.0 ± 10.6	54.1 ± 10.5
Hyperactivity	561	2.9	55.5 ± 10.8	55.6 ± 10.7
ADHD Index	560	3.1	54.1 ± 10.5	54.1 ± 10.4
CGI Restless-Impulsive	561	2.9	54.5 ± 10.3	54.6 ± 10.3
CRS-R DSM-IV
CRS: DSM IV Inattentive	561	2.9	53.3 ± 10.2	53.4 ± 10.2
CRS: DSM IV Hyperactive-Impulsive	561	2.9	57.1 ± 10.7	57.1 ± 10.6
CRS: DSM IV Total	561	2.9	55.4 ± 10.4	55.5 ± 10.3
Child characteristics
Blood lead level (μg/dL)	412	28.7	3.4 ± 2.9	3.4 ± 3.1
Children’s age (years)	578	0	9.1 ± 1.3	9.1 ± 1.3
Children’s sex, female	578	0	289 (50.0)	289 (50.0)
Birth weight (kg)	577	0.2	3.1 ± 0.4	3.1 ± 0.4
Parental characteristics
Maternal age (years)	578	0	26.0 ± 5.4	26.0 ± 5.4
Maternal education (years)	578	0	10.9 ± 2.8	10.9 ± 2.8
Mothers’ marital status, married	578	0	421 (72.8)	421 (72.8)
Smoking during pregnancy, smoked	577	0.2	18 (3)	18 (3)
Paternal education (years)	534	7.6	10.7 ± 3.5	10.6 ± 3.5
SES level index	553	4.3	8.7 ± 3.2	8.8 ± 3.3
SES, socioeconomic status. ^***a***^Behavior assessments were conducted at the follow-up visit when children were 6 to 13 years old. Children’s blood lead levels, parental information, and SES level index were assessed at the behavior assessment. SES level index was calculated by summarization of household assets at follow-up visit.				

**Figure 1:**
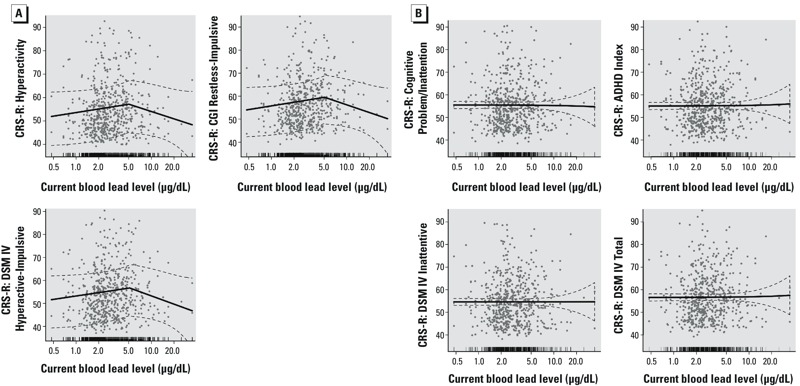
Adjusted associations (95% CIs; dashed lines) between a 1-μg/dL increase in blood lead and CRS-R outcomes in children 6–13 years of age using the imputed data set (*n* = 578). In each plot, the *x*-axis represents children’s blood lead levels (μg/dL) on a logarithmic scale; the *y*-axis represents the Conners Rating Scales-Revised (CRS-R) scores. The tick marks on the *x*-axis indicate the distribution of the observations. (*A*) Adjusted associations between blood lead and CRS-R hyperactive type outcomes from segmented regressions, with each estimated breakpoint and its 95% CI indicated as gray points and line above the *x*-axis. (*B*) Adjusted associations between blood lead and CRS-R inattentive and overall types outcomes from linear regressions.

Concurrent blood lead at the time of neurobehavioral testing had mean ± SD values of 3.4 ± 3.1 μg/dL. About 15% of subjects had blood lead levels exceeding 5 μg/dL. Blood lead showed a nonlinear relationship with a subset of CRS-R responses from the LOWESS plot (see Figure S1). After adjusting for covariates, the association between blood lead and CRS-R Hyperactivity, CRS-R CGI Hyperactive-Impulsive, and CRS-R DSM-IV Restless-Impulsive showed breakpoints near 5 μg/dL (Figure 1A, Table 2). Below the breakpoint, a 1-μg/dL increase in blood lead was associated with significantly higher scores for each of these outcomes, indicating an increase in symptoms with higher exposure (Table 2). However, the association reached a plateau after the breakpoint, such that a 1-μg/dL higher blood lead was not associated with higher scores for these outcomes (Table 2). The segmented regression approach did not find biologically meaningful breakpoints in the relationship of blood lead to CRS-R scales of Cognitive Problems/Inattention, ADHD Index, and CRS-R and DSM IV scales of Inattentive and Total. The associations between blood lead level and these responses, estimated with linear regression, were not significant after adjusting for covariates (Table 2, rows without breakpoints shown).

**Table 2:** Adjusted*^a^* associations between a 1-μg/dL increase in blood lead and CRS-R outcomes in children 6–13 years from segmented regressions or least-square regressions using the imputed data set (*n* = 578).

Outcome	Breakpoint^*b*^ (95% CI) (μg/dL)	Slope1 (95% CI)^*c*^	*p*	Slope2 (95% CI)	*p*
CRS-R
Cognitive Problem/Inattention	NA	–0.03 (–0.3, 0.2)	0.85	NA	NA
Hyperactivity	5.0 (2.4, 7.6)	1.2 (0.3, 2.0)	0.01	–0.3 (–0.8, 0.2)	0.26
ADHD Index	NA	0.02 (–0.2, 0.3)	0.86	NA	NA
CGI Restless-Impulsive	5.1 (2.7, 7.5)	1.2 (0.3, 2.0)	0.007	–0.3 (–0.7, 0.1)	0.15
CRS-R DSM-IV
Inattentive	NA	0 (–0.3, 0.3)	0.98	NA	NA
Hyperactive-Impulsive	5.2 (2.5, 7.8)	1.1 (0.2, 2.0)	0.02	–0.3 (–0.8, 0.1)	0.15
Total	NA	0.03 (–0.2, 0.3)	0.73	NA	NA
^***a***^All models were adjusted for maternal marital status, age, educational years, SES, and ever smoked during pregnancy, and the child’s age at behavioral testing, sex, and birth weight. ^***b***^Breakpoints were optimized from iterations in piecewise regressions using the imputed dataset. ^***c***^Biological meaningful breakpoints were not found in the models marked “NA”; the estimate shown reflects single slope for the entire range of the exposure distribution estimated using ordinary least-square regressions.					

The results of the sensitivity analyses showed relatively few, and expected, differences from our primary results shown in Table 2 that used imputed data. Upon inclusion of five low birth weight subjects, the effect estimates did not change. The models using the CDC level of concern for blood lead exposure (5 μg/dL) as the breakpoint for segmented regression of CRS-R Hyperactivity and CRS-R CGI Restless-Impulsive, CRS-R DSM IV Hyperactive-Impulsive (see Table S2) were similar to estimates using the breakpoints identified by the analysis (5.0, 5.1, 5.2 μg/dL, respectively). Associations between low blood lead levels and these outcomes also were similar when models were limited to children with known values of all model variables (*n* = 362), including models that used the same breakpoints as in the primary model, and when all breakpoints were set to 5.0 μg/dL (see Tables S3 and S5, respectively). The association was similar when applying breakpoints estimated in the complete-case data only for blood lead levels ≤ 5.0 μg/dL; however, for blood lead levels > 5 μg/dL, the estimates were more negative but nonsignificant (see Table S4). Additionally, estimates were consistent with those shown in Table 2 for blood lead levels ≤ 5.0 μg/dL when based only on 342 samples measured at the University of Michigan. Sensitivity analyses of outcomes evaluated using linear regression (CRS-R Cognitive scale and ADHD Index, CRS-R DSM-IV Inattentive and Total scales) were consistent with those shown in Table 2 when based on complete-case data (see Table S3) and samples measured at the University of Michigan (see Table S6). Finally, estimates were similar for all outcomes when five children with low birth weight (< 2,000 g) were included in analyses using imputed data and model-derived breakpoints for segmented regressions (data not shown).

## Discussion

We found that concurrent blood lead was associated with hyperactive and impulsive behaviors in 6- to 13-year-old Mexican children. However, the association did not exhibit a linear relationship. We observed a trend of worsening responses associated with increasing blood lead levels below approximately 5 μg/dL, followed by a plateau or slightly decreasing trend when blood lead level was > 5 μg/dL. We did not observe any association between concurrent blood lead and inattentiveness or overall ADHD symptoms in these children.

ADHD is a psychiatric disorder with prevalence rising from 2.8% to 4.4% between 1998 and 2009 in 5- to 17-year-old Mexican-American children in the United States (Akinbami et al. 2011). Children with ADHD are primarily found to have impairments in attention, executive function, state regulation, motivation, and processing of temporal information (Nigg 2005). However, ADHD is highly heterogeneous and further classified into three subtypes that exhibit distinct behavior patterns: inattentive subtype (ADHD-I), hyperactive subtype (ADHD-H), and combined subtype (ADHD-C). For example, ADHD-C children typically demonstrate sluggish and hypoactive behavior, whereas ADHD-I children tend to be shy yet active (Carlson and Mann 2002; McBurnett et al. 2001). These distinct characteristics suggest that the underlying brain function alterations and associated etiologies of these ADHD subtypes may be different. Twin studies also support this idea, indicating high heritability of inattention, but not of hyperactivity or impulsivity (Greven et al. 2011; Willcutt et al. 2000a). However, the studies conducted in young twins could lead to overestimation of genetic influence due to shared living environments. Recent studies have shown mixed findings in the associations between lead exposure and the subtypes of ADHD (Goodlad et al. 2013). There is increasing evidence of a link between lead exposure and hyperactive behavior (Boucher et al. 2012b; Hong et al. 2015; Nigg 2008; Nigg et al. 2010a; Sioen et al. 2013). Our finding that, among children with blood lead levels ≤ 5.0 μg/dL, concurrent blood lead is associated with hyperactive and impulsive behavior, but not inattention or combined subtypes of behavior suggests a potential environmental etiology of ADHD-H, which genetics alone cannot fully explain.

The dopamine pathway is one of the major neurotransmitter pathways involved in ADHD (Bellgrove et al. 2005; Carrasco et al. 2004; Hamarman et al. 2004; Henríquez et al. 2008; Swanson et al. 2000). The association between lead and ADHD symptoms in our study population, specifically with hyperactivity-impulsivity, may reflect effects of lead on the dopamine system (Cory-Slechta 1995, 1997; Luo et al. 2014). Dopaminergic neurons are especially susceptible to oxidative stress due to the reactive oxygen species generated during dopamine degradation process (Meiser et al. 2013; Miyazaki and Asanuma 2008; Sulzer and Zecca 2000). Lead may accelerate dopaminergic neuron loss by imposing oxidative stress (Chetty et al. 2005; Hsu 1981; Lawton and Donaldson 1991; Sulzer 2007). Lead also disrupts homeostasis of calcium-dependent neurotransmitters, such as dopamine (Simons 1993), by competing with calcium at its binding sites (Goering 1993) and inhibits dopamine transporter activity (Luo et al. 2014).

Lead exposure has previously been reported to be associated with ADHD or ADHD symptoms. Higher blood lead levels have been observed in children with ADHD (Froehlich et al. 2009; Kim et al. 2013; Sánchez-Villegas et al. 2014; Wang et al. 2008), and positive associations between blood lead and increased likelihood of ADHD symptoms or ADHD diagnosis have been observed in studies using various behavioral scales in cross-sectional studies (Froehlich et al. 2009; Ha et al. 2009; Nicolescu et al. 2010). The few studies that have explored ADHD subtypes suggest that lead exposure is likely to result in hyperactivity, impulsivity (Boucher et al. 2012a; Cho et al. 2010; Nigg et al. 2008, 2010b) and impaired executive functions (Canfield et al. 2003b; Roy et al. 2009). Most published studies with low lead exposure levels (mean blood lead levels ≤ 5 μg/dL) in the past decade have shown that lead is associated with ADHD at levels between 2 to 4 μg/dL measured in whole blood in children between 3 and 17 years (Braun et al. 2006; Cho et al. 2010; Froehlich et al. 2009; Ha et al. 2009; Kim et al. 2010; Nigg et al. 2008, 2010b). A few studies with higher lead exposure levels (mean blood lead levels ≥ 10 μg/dL) in the study population showed the association when blood lead levels are > 10 μg/dL (Roy et al. 2009; Sánchez-Villegas et al. 2014; Wang et al. 2008).

Our study is consistent with most of the existing literature suggesting that the association between lead and ADHD-symptoms occurs at levels ≤ 5 μg/dL (Braun et al. 2006; Cho et al. 2010; Froehlich et al. 2009; Ha et al. 2009; Kim et al. 2010; Nigg et al. 2008, 2010b). Our data showed a clear pattern of higher (worse) scores on hyperactivity and restless-impulsive behaviors associated with increasing blood lead ≤ 5 μg/dL. As blood lead rose > 5 μg/dL, the data did not exhibit continuing increasing trend on these scales, perhaps because 85% of subjects in our data had blood lead levels ≤ 5 μg/dL and only 2.7% of subjects had blood lead levels > 10 μg/dL. This exposure distribution underpowered our ability to interpret the dose–response relationship at the higher exposure range.

Our study has several limitations. First, ADHD behavior is a complex phenotype. The mechanism of ADHD inattentive subtypes is still not comprehensively understood. For instance, deficits in executive function and motivation processes that are observed in ADHD are not specific to ADHD (Kuntsi et al. 2004; Nigg 2005). Additionally, attention processes are mediated by cognitive function. Therefore, the null findings in the association of lead with inattentive symptoms in our study may not directly reflect on inattentive behavior problem per se. It is worth noting that most of the association studies of lead and cognition have shown a link between declined cognitive functions and lead exposure at low (≤ 5 μg/dL) and higher levels (i.e., Canfield et al. 2003a; Chandramouli et al. 2009; Lanphear et al. 2005). However, our inference on the lead exposure–ADHD symptoms dose–response relationship is limited due to lack of observation at higher exposure range. Further analyses are required to disentangle the intermediate effects of higher brain functioning involved in cognitive processes from those involved in attention processes. Also, as previously mentioned, genetics likely plays a major role in ADHD. In this analysis, we examined only the main effect of lead exposure without available information on family history or genetic markers linked to ADHD. Additionally, even though we used the CRS-R Spanish version in our study, this questionnaire may not be entirely culturally appropriate. This may be reflected by our data demonstrating higher means than expected in the CRS-R scales (Conners 2000). We also encountered difficulties in obtaining CRS-R evaluations from teachers, which could be less subjective than parental ratings. However, mothers were blinded to their children’s blood lead test results while evaluating the children’s ADHD symptoms. It is unlikely that their ratings were informatively biased according to the children’s concurrent blood lead levels. Nevertheless, the information bias that may occur in mothers’ response cannot be completely dismissed. Similarly, HOME [Home Observation for Measurement of the Environment (Caldwell and Bradley 1984)] inventory scores were only available in a subset of 325 households. However, we compared the subjects with and without HOME score and found no significant differences in the population characteristics (data not shown). We performed a multivariable linear regression analysis and found that variables we used for adjustment in our main analysis collectively explained 96.1% of variations in the HOME score. The demographic characteristics in our study population could also constrain our ability to explore lead effect in different subpopulations. For example, only 18 mothers (3%) reported having smoked during pregnancy, which limited our ability to assess the potential modifying effect of smoking during pregnancy on the associations between lead and ADHD symptoms. The socioeconomic class in our population also had a narrow range. This limits the generalizability of our findings in relation to the populations that are at the highest or lowest ends of the socioeconomic spectrum, and may limit the power to detect the interactions between lead exposure and socioeconomic class on ADHD-like outcomes. We recommend further study on effect of lead with regard to these aspects. The lead exposure data were obtained from two laboratories, which could result in varying degrees of measurement error across samples; however, our between-lab cross validation showed high consistency. We also encountered a fair number of subjects declining blood draw for blood lead measures, which confined our observations of exposure. We used imputation to fill in missing values, and assumed values were missing at random in our imputation models because declining phlebotomy is unlikely correlated to the blood lead level. Comparing our analyses with imputed data to the complete case analysis, we observed slight differences in estimated associations. This may be attributable to the fact that removing subjects with missing values from the analysis assumes data are missing completely at random; this is a strong assumption and is unlikely to be met because those who declined phlebotomy tended to be younger. Finally, our analysis examined the cross-sectional association between lead measured at the same time as the behavioral assessment. Future studies should examine the independent effect of perinatal lead exposures and their potential modifying role on the effect of concurrent exposures. There are few studies that have linked perinatal lead exposures to ADHD in childhood (Kim et al. 2013; Yolton et al. 2014). It could also be that other measures of exposure, such as cumulative lead levels or blood lead measured at other time points, may be more etiologically relevant.

Nonetheless, our study has the advantages of a relatively large sample size, comprehensive evaluation of socioeconomic status, and estimates of maternal parenting behavior. We took advantage of the longitudinal design of the parent study by including maternal cigarette smoking during pregnancy and infant birth weight in the models to control for these potentially confounding effects and to exclude any indirect effect of prenatal lead exposure that can be reflected on birth weight (Nishioka et al. 2014). Our study also has a good representation of a low- to middle-class urban Mexican population and accurate estimate of concurrent lead exposure. Our findings also encourage additional explorations on the associations between lead exposure and other behavior problems, such as delinquent behavior and violence, which could have more serious impacts to the society and public health.

## Conclusions

In summary, our study concurs with previous studies demonstrating an association of concurrent lead exposure with ADHD-symptoms. In particular, we observed an association between lead and hyperactivity and impulsivity behaviors when concurrent blood lead was ≤ 5 μg/dL. However, this association was attenuated when levels were > 5 μg/dL, which may explain why behavior problems are not often linked with lead exposure on an individual basis, given the lack of a clear dose–response relationship at levels more typically characterized as “elevated.” The potential impact of low-level lead exposure on a population basis is large and raises a serious public health concern particularly in areas where environmental lead contamination continues.

## Supplemental Material

(643 KB) PDF
