# Supplementary material for: Childhood Blood Lead Levels and Symptoms of Attention Deficit Hyperactivity Disorder (ADHD): A Cross-Sectional Study of Mexican Children
Source: Environ Health Perspect. 2015 Dec 8;124(6):868–74. doi: 10.1289/ehp.1510067 (PMC4892926; doi:10.1289/ehp.1510067)
Supplement: (643 KB) PDF [file ehp.1510067.s001.acco.pdf]

Environ Health Perspect
DOI: 10.1289/ehp.1510067

Note to readers with disabilities: *EHP* strives to ensure that all journal content is accessible to all readers. However, some figures and Supplemental Material published in *EHP* articles may not conform to 508 standards due to the complexity of the information being presented. If you need assistance accessing journal content, please contact ehp508@niehs.nih.gov. Our staff will work with you to assess and meet your accessibility needs within 3 working days.

Supplemental Material

Childhood Blood Lead Levels and Symptoms of Attention Deficit Hyperactivity Disorder (ADHD): A Cross-Sectional Study of Mexican Children

Siying Huang, Howard Hu, Brisa N Sánchez, Karen E. Peterson, Adrienne S. Ettinger, Héctor Lamadrid-Figueroa, Lourdes Schnaas, Adriana Mercado-García, Robert O. Wright, Niladri Basu, David E. Cantonwine, Mauricio Hernández-Avila, and Martha María Téllez-Rojo

Table of Contents

Table S1. Adjusted associations between a 1-µg/dL increase in blood lead and CRS-R outcomes in children age 6 to 13 years using segmented regression with different initial values for iterations

Table S2. Adjusted associations between a 1-µg/dL increase in blood lead and CRS-R outcomes in children age 6 to 13 years from piecewise linear regressions using 5µg/dL as the breakpoint in imputed dataset (N=578)

Table S3. Adjusted associations between a 1-µg/dL increase in blood lead and CRS-R outcomes in children age 6 to 13 years from piecewise linear regressions or simple linear regressions using complete-case data (N=362)

Table S4. Adjusted associations between a 1-µg/dL increase in blood lead and CRS-R outcomes in children age 6 to 13 years from piecewise linear regressions with data-driven breakpoints in the complete-case data (N=361)

Table S5. Adjusted associations between a 1-µg/dL increase in blood lead and CRS-R outcomes in children age 6 to 13 years from piecewise linear regressions using 5µg/dL as the breakpoint in complete-case data (N=362)

Table S6. Adjusted associations between a 1-µg/dL increase in blood lead and CRS-R outcomes in children age 6 to 13 years from piecewise linear regressions or simple linear regressions using the samples measured from University of Michigan department of Environmental Health Sciences labs only (N=342)

Figure S1. Crude associations between a 1-µg/dL increase in blood lead and CRS-R outcomes in children aged 6 to 13 years with locally weighted scatterplot smoothing (LOWESS) method. The X-axis represents children's blood lead levels (µg/dL) and is converted into logarithmic scale; the Y-axis represents the Conners Rating Scales-Revised (CRS-R) scores.

Table S1. Adjusted [a] associations between a 1-µg/dL increase in blood lead and CRS-R outcomes in children age 6 to 13 years using segmented regression with different initial values for iterations

Outcome [b]	Initial break-points	Breakpoint estimates (95%CI)	Slope1 (95%CI)	Slope2 (95%CI)
Cognitive	1	0.85 (0.53, 1.18)	-24.03 (-140.7, 92.62)	0 (-0.27, 0.27)
Problem/Inattention	1	0.85 (0.53, 1.18)	-24.03 (-140.7, 92.62)	0 (-0.27, 0.27)
	8	0.87 (0.49, 1.25)	-22.33 (-139, 94.33)	0 (-0.27, 0.28)
	8	1.34 (0.01, 2.68)	-3.88 (-19.13, 11.38)	0 (-0.28, 0.28)
	15	0.86 (0.52, 1.20)	-23.55 (-140.2, 93.10)	0 (-0.27, 0.28)
	15	0.88 (0.46, 1.31)	-21.08 (-137.8, 95.62)	0 (-0.27, 0.28)
**Hyperactivity**	1	5.02 (2.42, 7.63)	1.15 (0.25, 2.05)	-0.29 (-0.81, 0.22)
	1	5.02 (2.42, 7.62)	1.15 (0.25, 2.05)	-0.29 (-0.80, 0.22)
	8	5.04 (2.43, 7.66)	1.15 (0.25, 2.04)	-0.3 0(-0.81, 0.21)
	8	5.02 (2.42, 7.62)	1.15 (0.25, 2.05)	-0.29 (-0.80, 0.22)
	15	5.02 (2.42, 7.62)	1.15 (0.25, 2.05)	-0.29 (-0.80, 0.22)
	15	5.03 (2.42, 7.63)	1.15 (0.25, 2.05)	-0.29 (-0.81, 0.22)
ADHD Index	1	20.54 (-22.21, 63.28)	0.15 (NA, NA)	-0.57 (NA, NA)
	1	6.04 (-1.75, 13.84)	0.37 (-0.39, 1.14)	-0.17 (-0.65, 0.31)
	8	20.41 (-23.11, 63.93)	0.15 (NA, NA)	-0.57 (NA, NA)
	8	5.32 (-1.03, 11.66)	0.44 (-0.41, 1.29)	-0.16 (-0.62, 0.31)
	15	5.69 (-1.16, 12.55)	0.42 (-0.38, 1.22)	-0.17 (-0.65,0.31)
	15	20.52 (-22.35, 63.38)	0.15 (NA, NA)	-0.57 (NA, NA)
**CGI Restless-Impulsive**	1	5.08 (2.7, 7.46)	1.19 (0.33, 2.04)	-0.31 (-0.73, 0.11)
	1	5.09 (2.71, 7.47)	1.19 (0.33, 2.04)	-0.31 (-0.73, 0.11)
	8	5.06 (2.70, 7.43)	1.19 (0.33, 2.05)	-0.31 (-0.73, 0.10)
	8	5.09 (2.71, 7.47)	1.19 (0.33, 2.04)	-0.31 (-0.73, 0.11)
	15	5.08 (2.70, 7.45)	1.19 (0.33, 2.05)	-0.31 (-0.73, 0.11)
	15	5.08 (2.70, 7.46)	1.19 (0.33, 2.04)	-0.31 (-0.73, 0.11)
DSM IV Inattentive	1	0.83 (0.57, 1.09)	-28.85 (-168.20, 110.50)	0.03 (-0.24, 0.30)
	1	0.83 (0.57, 1.09)	-28.85 (-168.20, 110.50)	0.03 (-0.24, 0.30)
	8	0.89 (0.50, 1.28)	-22.98 (-135.60, 89.68)	0.03 (-0.24, 0.30)
	8	0.98 (0.48, 1.47)	-15.26 (-64.98, 34.45)	0.04 (-0.23, 0.31)
	15	0.89 (0.50, 1.28)	-23.06 (-135.70, 89.61)	0.03 (-0.24, 0.30)
	15	0.89 (0.50, 1.27)	-23.21 (-135.90, 89.46)	0.03 (-0.24, 0.30)
**DSM IV Hyperactive-**	1	5.13 (2.47, 7.78)	1.09 (0.19, 2.00)	-0.33 (-0.80, 0.13)
**Impulsive**	1	5.14 (2.47, 7.81)	1.09 (0.19, 1.99)	-0.33 (-0.80, 0.13)
	8	5.13 (2.47, 7.79)	1.09 (0.19, 1.99)	-0.33 (-0.80, 0.13)
	8	5.13 (2.47, 7.79)	1.09 (0.19, 1.99)	-0.33 (-0.80, 0.13)
	15	5.12 (2.47, 7.78)	1.09 (0.19, 2.00)	-0.33 (-0.80, 0.13)
	15	5.12 (2.47, 7.78)	1.09 (0.19, 2.00)	-0.33 (-0.80, 0.13)
DSM IV Total	1	5.47 (0.71, 10.23)	0.59 (-0.23, 1.40)	-0.22 (-0.71, 0.26)
	1	5.27 (0.73, 9.81)	0.6 (-0.25, 1.46)	-0.21 (-0.68, 0.25)
	8	5.46 (0.71, 10.22)	0.59 (-0.23, 1.40)	-0.22 (-0.71, 0.26)
	8	5.47 (0.71, 10.23)	0.59 (-0.23, 1.40)	-0.22 (-0.71, 0.26)
	15	5.47 (0.71, 10.23)	0.59 (-0.23, 1.40)	-0.22 (-0.71, 0.26)
	15	5.61 (0.60, 10.61)	0.57 (-0.22, 1.36)	-0.23 (-0.71, 0.26)

a. All models adjusted for maternal marital status, age, educational years, and socioeconomic status, ever smoked during pregnancy, and the child's age at behavioral testing, sex and birth weight. NA: Not applicable; confidence interval could not be derived by the algorithm. Distribution of blood lead level in µg/dL: min= 0.5, 1st quartile= 1.9, mean= 3.4, 3rd quartile= 4.0, max= 34.8. We kept two digits after the decimal point due to that the most of differences occurred at this level.

b. The variables in bold showed stable breakpoint estimates and biological meaningful effect estimates of blood lead and behavior associations.

Table S2. Adjusted [a] associations between a 1-µg/dL increase in blood lead and CRS-R outcomes in children age 6 to 13 years from piecewise linear regressions using 5µg/dL as the breakpoint in imputed dataset (N=578) [b]

Outcome	Breakpoint (µg/dL)	Slope1 (95%CI)	p	Slope2 (95%CI)	p
CRS-R					
Hyperactivity	5.0	1.2 (0.4, 2.0)	0.002	-0.3 (-0.7, 0.1)	0.14
CGI Restless-Impulsive	5.0	1.2 (0.4, 2)	0.001	-0.3 (-0.7, 0.1)	0.09
CRS-R DSM-IV					
Hyperactive-Impulsive	5.0	1.1 (0.3, 1.9)	0.003	-0.3 (-0.7, 0.1)	0.10

a. All models adjusted for maternal marital status, age, educational years, and socioeconomic status, ever smoked during pregnancy, and the child's age at behavioral testing, sex and birth weight.
b. Biological meaningful breakpoints were not found in the rest of models and results were not included.

Table S3. Adjusted [a] associations between a 1-μg/dL increase in blood lead and CRS-R outcomes in children age 6 to 13 years from piecewise linear regressions or simple linear regressions using complete-case data (N=362)

Outcome	Breakpoint [b] (μg/dL)	Slope1 (95%CI) [c]	p	Slope2 (95%CI)	p
CRS-R					
Cognitive Problem/Inattention	NA	-0.2 (-0.6, 0.2)	0.27	NA	NA
Hyperactivity	5.0	1.0 (0.1, 2.0)	0.04	-0.3 (-0.8, 0.2)	0.31
ADHD Index	NA	-0.1 (-0.5, 0.3)	0.63	NA	NA
CGI Restless-Impulsive	5.1	1.0 (0.1, 1.9)	0.03	-0.5 (-1.0, 0.1)	0.08
CRS-R DSM-IV					
Inattentive	NA	-0.1 (-0.5, 0.3)	0.57	NA	NA
Hyperactive-Impulsive	5.2	1.0 (0.0, 1.9))	0.04	-0.4 (-0.9, 0.1)	0.13
Total	NA	-0.06 (-0.4, 0.3)	0.73	NA	NA

a. All models adjusted for maternal marital status, age, educational years, and socioeconomic status, ever smoked during pregnancy, and the child's age at behavioral testing, sex and birth weight.
b. Breakpoints were optimized from iterations in piecewise regressions using the imputed dataset, as shown in Table 2.
c. Biological meaningful breakpoints were not found in the models marked as "NA" in the breakpoint column; the estimate shown reflects single slope for the entire range of the exposure distribution estimated using ordinary least square regressions.

Table S4. Adjusted [a] associations between a 1-µg/dL increase in blood lead and CRS-R outcomes in children age 6 to 13 years from piecewise linear regressions with data-driven breakpoints in the complete-case data (N=361 [b])

Outcome	Breakpoint [c] (95%CI) (µg/dL)	Slope1 (95%CI)	p	Slope2 (95%CI)	p
CRS-R					
Hyperactivity	5.1 (2.7, 7.6)	1.3 (0.1, 2.4)	0.04	-0.9 (-2, 0.1)	0.09
CGI Restless-Impulsive	5.1 (2.5, 7.8)	1.1 (0.0, 2.3)	0.05	-0.8 (-1.7, 0.0)	0.06
CRS-R DSM-IV					
Hyperactive-Impulsive	5.2 (2.6, 7.7)	1.2 (0.0, 2.4)	0.04	-0.9 (-1.9, 0.1)	0.08

a. All models adjusted for maternal marital status, age, educational years, and socioeconomic status, ever smoked during pregnancy, and the child's age at behavioral testing, sex and birth weight.
b. One outlier (>4SD) on blood lead levels was excluded from the data.
c. Breakpoints were optimized from iterations in piecewise regressions using the complete-case dataset. Biological meaningful breakpoints were not found in the rest of models and results were not included.

Table S5. Adjusted [a] associations between a 1-µg/dL increase in blood lead and CRS-R outcomes in children age 6 to 13 years from piecewise linear regressions using 5µg/dL as the breakpoint in complete-case dataset (N=362) [b]

**Outcome**	**Breakpoint (µg/dL)**	**Slope1 (95%CI)**	**p**	**Slope2 (95%CI)**	**p**
CRS-R					
Hyperactivity	5.0	1.0 (0.0, 2.0)	0.04	-0.3 (-0.8, 0.2)	0.31
CGI Restless-Impulsive	5.0	1.0 (0.0, 2.0)	0.03	-0.4 (-0.9, 0.0)	0.08
CRS-R DSM-IV					
Hyperactive-Impulsive	5.0	1.0 (0.0, 2.0)	0.04	-0.4 (-0.9, 0.1)	0.14

a. All models adjusted for maternal marital status, age, educational years, and socioeconomic status, ever smoked during pregnancy, and the child's age at behavioral testing, sex and birth weight.
b. Biological meaningful breakpoints were not found in the rest of models and results were not included.

Table S6. Adjusted [a] associations between a 1-μg/dL increase in blood lead and CRS-R outcomes in children age 6 to 13 years from piecewise linear regressions or simple linear regressions using the samples measured from University of Michigan department of Environmental Health Sciences labs only (N=342)

**Outcome**	**Breakpoint (μg/dL)** [b]	**Slope1 (95%CI)** [c]	**p**	**Slope2 (95%CI)**	**p**
CRS-R					
Cognitive Problem/Inattention	NA	-0.1 (-0.5, 0.2)	0.43	NA	NA
Hyperactivity	5.0	1.0 (0.1, 2.0)	0.03	-0.2 (-0.7, 0.3)	0.44
ADHD Index	NA	-0.1 (-0.5, 0.3)	0.61	NA	NA
CGI Restless-Impulsive	5.0	0.9 (0.0, 1.8)	0.04	-0.4 (-0.8, 0.1)	0.14
CRS-R DSM-IV					
Inattentive	NA	-0.04 (-0.4, 0.3)	0.84	NA	NA
Hyperactive-Impulsive	5.0	1.0 (0.1, 1.9)	0.03	-0.3 (-0.9, 0.2)	0.19
Total	NA	0 (-0.4, 0.4)	0.98	NA	NA

a. All models adjusted for maternal marital status, age, educational years, and socioeconomic status, ever smoked during pregnancy, and the child's age at behavioral testing, sex and birth weight.
b. Breakpoints could not be estimated in the UMEHS samples and were fixed at 5μg/dL.
c. Biological meaningful breakpoints were not found in the models marked as "NA" in the breakpoint column; the estimate shown reflects single slope for the entire range of the exposure distribution estimated using ordinary least square regressions.

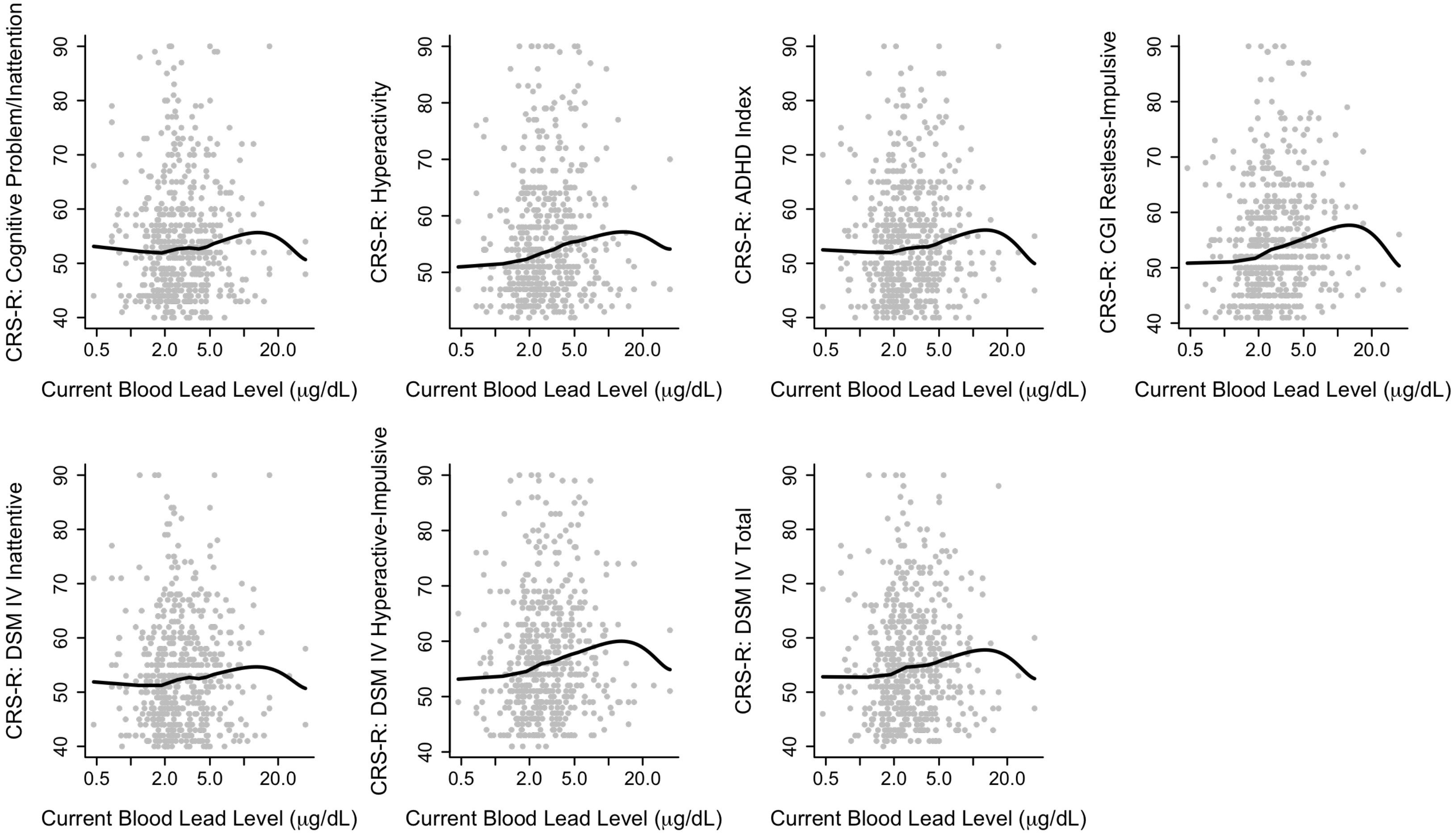

Figure S1. Crude associations between a 1-μg/dL increase in blood lead and CRS-R outcomes in children aged 6 to 13 years with locally weighted scatterplot smoothing (LOWESS) method. The X-axis represents children's blood lead levels (μg/dL) and is converted into logarithmic scale; the Y-axis represents the Conners Rating Scales-Revised (CRS-R) scores.
